# Metabonomic Characteristics of Myocardial Diastolic Dysfunction in Type 2 Diabetic Cardiomyopathy Patients

**DOI:** 10.3389/fphys.2022.863347

**Published:** 2022-05-09

**Authors:** Mingyu Hao, Jianxin Deng, Xiaohong Huang, Haiyan Li, Huiting Ou, Xiangsheng Cai, Jiajie She, Xueting Liu, Ling Chen, Shujuan Chen, Wenlan Liu, Dewen Yan

**Affiliations:** ^1^ Department of Endocrinology, Shenzhen Clinical Research Center for Metabolic Diseases, Shenzhen Second People’s Hospital, The First Affiliated Hospital of Shenzhen University, Health Science Center of Shenzhen University, Shenzhen, China; ^2^ Shenzhen Institutes of Advanced Technology, Chinese Academy of Sciences, Shenzhen, China; ^3^ Institute of Translational Medicine, University of Chinese Academy of Science-Shenzhen Hospital, Shenzhen, China; ^4^ The First Affiliated Hospital of Shenzhen University, Reproductive Medicine Centre, Shenzhen Second People’s Hospital, Shenzhen, China; ^5^ Department of Neurosurgery, Shenzhen Key Laboratory of Neurosurgery, Shenzhen Second People’s Hospital, Shenzhen University First Affiliated Hospital, Shenzhen, China

**Keywords:** diabetic cardiomyopathy (DCM), metabolic profiles, metabonomics, diabetes, serum metabolites

## Abstract

Diabetic cardiomyopathy (DCM) is one of the most essential cardiovascular complications in diabetic patients associated with glucose and lipid metabolism disorder, fibrosis, oxidative stress, and inflammation in cardiomyocytes. Despite increasing research on the molecular pathogenesis of DCM, it is still unclear whether metabolic pathways and alterations are probably involved in the development of DCM. This study aims to characterize the metabolites of DCM and to identify the relationship between metabolites and their biological processes or biological states through untargeted metabolic profiling. UPLC-MS/MS was applied to profile plasma metabolites from 78 patients with diabetes (39 diabetes with DCM and 39 diabetes without DCM as controls). A total of 2,806 biochemical were detected. Compared to those of DM patients, 78 differential metabolites in the positive-ion mode were identified in DCM patients, including 33 up-regulated and 45 down-regulated metabolites; however, there were only six differential metabolites identified in the negative mode including four up-regulated and two down-regulated metabolites. Alterations of several serum metabolites, including lipids and lipid-like molecules, organic acids and derivatives, organic oxygen compounds, benzenoids, phenylpropanoids and polyketides, and organoheterocyclic compounds, were associated with the development of DCM. KEGG enrichment analysis showed that there were three signaling pathways (metabolic pathways, porphyrin, chlorophyll metabolism, and lysine degradation) that were changed in both negative- and positive-ion modes. Our results demonstrated that differential metabolites and lipids have specific effects on DCM. These results expanded our understanding of the metabolic characteristics of DCM and may provide a clue in the future investigation of reducing the incidence of DCM. Furthermore, the metabolites identified here may provide clues for clinical management and the development of effective drugs.

## Introduction

Heart failure (HF) is associated with high mortality and morbidity. About 20% of HF patients are diagnosed with complications of type 2 diabetes mellitus (T2DM) (Formiga et al., 2020). In 1972, Rubber et al. proposed that diabetic patients could experience cardiomyopathy without coronary artery ischemia (Rubler et al., 1972). People now have a better knowledge of this particular cardiac ailment, thanks to decades of research. Diabetic cardiomyopathy (DCM) is defined by the European Society of Cardiology as cardiomyopathy with myocardial structural changes and ventricular systolic and diastolic dysfunction in patients with diabetes, excluding hypertensive heart disease, coronary heart disease, and cardiovascular disease (Authors/Task Force et al., 2013). DCM is one of the most common cardiovascular complications in patients with diabetes. According to epidemiological statistics, the prevalence rate of diabetes and diabetic cardiomyopathy in the general population is 16.9 and 1.1%, respectively. The mortality rate of patients with diabetic cardiomyopathy is 31% (Dandamudi et al., 2014). In the early stages, DCM is characterized by left ventricular hypertrophy, increased myocardial stiffness, increased ventricular filling pressure, and impaired diastolic function. In the late stage, cardiac fibrosis is aggravated, the diastolic function is further damaged, and secondary is accompanied by systolic dysfunction (Westermeier et al., 2016; Gilca et al., 2017). DCM progresses slowly and can only be diagnosed when the heart shows some degree of dysfunction, one of the important causes of death in patients with diabetes (Dandamudi et al., 2014). Therefore, HF with preserved ejection fraction (HFpEF) is regarded as the clinical feature of DCM. The disturbance of cardiomyocyte active relaxation can cause a decrease in coronary blood perfusion, induce myocardial fibrosis and myocardial remodeling, make the ventricle stiff, decrease compliance and further aggravate the diastolic cardiac dysfunction, and develop into left ventricular ejection fraction preserved HF (Park, 2021). At present, the clinical diagnosis of DCM is still challenging because its pathogenesis is not well understood, and there is a lack of reliable and specific clinical diagnostic markers in the early stages of the disease. It is urgently needed to explore further biomarkers that can be used to recognize early DCM.

At present, some serum biomarkers are considered to have a certain predictive value in diagnosing DCM. Natriuretic peptides can be used to identify the changes in early diastolic function, which can predict the risk of cardiovascular complications in people with poor blood glucose control. Still, its positive predictive value is poor (Romano et al., 2010). Shaver et al. found that the level of serum adiponectin in patients with uncomplicated diabetes was lower than that in normal subjects, and the level of adiponectin in patients with DCM was further decreased, suggesting that monitoring serum adiponectin may help with the diagnosis of early cardiomyopathy in patients with diabetes (Shaver et al., 2016). Ana Lorenzo-Almorós et al. found that Galectin-3 is associated with an increased risk of cardiovascular events in diabetic patients with stable CAD; galectin-3 may work as a diagnostic and prognostic biomarker (Lorenzo-Almoros et al., 2020). Another study reported that NT-proBNP is consistently related with reducing cardiac function in euglycemia. The prospective value of NT-proBNP was superior to galectin-3 in assessing reduced systolic and diastolic function in patients without T2DM (Schmitt et al., 2021). Cardiac troponin I (cTnI) is a clinically sensitive and particular marker of myocardial injury (Liu et al., 2015). In diabetic patients, the level of serum cTnI in the DCM group is higher than that in the diabetic group, which is consistent with those of the experimental animal model of DCM, where the level of cTnI in the plasma of T2DM mice with heart failure is significantly increased (Korkmaz-Icoz et al., 2016). However, cTnI as a biological indicator of DCM lacks enough experimental evidence, so it can only be used as an auxiliary indicator. Akbal et al. found elevated heart-type fatty acid-binding protein (H-FABP) levels in T2DM patients with early myocardial injury suggesting that this factor may contribute to the recognition of early DCM (Akbal et al., 2009). TGF-β can promote myocardial fibrosis and myocardial hypertrophy. Tan et al. used a TGF-β inhibitor in the T2DM mouse model and found that the ventricular diastolic disturbance in mice was improved (Tan et al., 2012). TGF-β is expected to become an important marker for the diagnosis and treatment of DCM. Ihm et al. studied the correlation between the increase of type I procollagen N-terminal peptide (PINP) and the echocardiographic evidence of myocardial diastolic dysfunction (Ihm et al., 2007). They revealed that the left ventricular diastolic function was related to serum PINP in patients with early T2DM. However, there is a lack of clear diagnostic criteria for DCM, and the inclusion criteria for DCM patients were not the same in these clinical studies. At the same time, related serum biomarkers often involve a variety of pathophysiological mechanisms and often appear in other diseases and other complications of diabetes at the same time. It is still urgent to further explore the disease mechanism and find more specific biomarkers for the early diagnosis of DCM.

Metabolomics provides a way to analyze all metabolites in organisms quantitatively and determine the relative relationship between metabolites and their physiological and pathological changes (Mishra et al., 2017). The untargeted metabolomics compares the experimental and control groups to detect all metabolites in a sample and discover statistically significant differences in metabolites between different groups which may explain the relationship between metabolites and biological process states (Tan et al., 2020).

T2DM patients were recruited from the Department of Endocrinology at the First Affiliated Hospital of Shenzhen University for this study. The metabolites of T2DM patients with cardiac diastolic dysfunction were quantitatively analyzed by metabolomics, and bioinformatic and statistical methods screened the differential metabolites. A total of 2,806 biochemicals were detected, including lipids and lipid-like molecules, organic acids and derivatives, organic oxygen compounds, benzenoids, phenylpropanoids, polyketides, and organoheterocyclic compounds, were associated with the development of DCM. Our findings contributed to a better understanding of the metabolic characteristics of DCM. They may provide a clue in future research to reduce the incidence of DCM and its progression to HFpEF.

## Materials and Methods

### Subjects and Grouping

This research explores the differences in metabolites between T2DM patients with or without diastolic dysfunction, and the diagnosis of T2DM according to 2021ADA-Standards of Medical Care in Diabetes: FPG ≥126 mg/dl (7.0 mmol/L) or 2-h PG ≥ 200 mg/dl (11.1 mmol/L) during OGTT or A1C ≥ 6.5% (48 mmol/mol) or in a patient with classic symptoms of hyperglycemia or hyperglycemic crisis, random plasma glucose ≥200 mg/dl (11.1 mmol/L). This study enrolled T2DM inpatients at the Department of Endocrinology, the First Affiliated Hospital of Shenzhen University, from January 2021 to May 2021, excluding other types of diabetes. The patients diagnosed with a combination of hypertension, coronary heart disease, thyroid disease, chronic kidney disease, rheumatic heart disease, primary cardiomyopathy, and congestive heart failure were excluded. According to Doppler echocardiography, LVEF ≥50% was defined as patients with preserved ejection fraction. The patients in the control group had normal diastolic function. The age (46.49 ± 1.63 vs. 59.41 ± 1.74) and the disease course (8.18 ± 1.08 vs. 12.77 ± 1.18), the male to female ratio is 2:1. The procedure followed in this study is in line with the standards established by the Human Trial Committee of Shenzhen Second People’s Hospital, approved by the ethics committee, ethics number is 20220210001, and signed informed consent for clinical research with all subjects.

### Biochemical Index Detection

The patient’s age, body mass index (BMI), height, and other clinical information were obtained through medical history inquiry and physical examination. Early in the morning, fasting venous blood was collected. An automatic biochemical analyzer was used to detect fasting blood glucose (FBG), 2-h postprandial blood glucose (PBG), and glycosylated hemoglobin (HbA1c). Serum triglycerides (TG), total cholesterol (TC), low-density lipoprotein (LDL-C), high-density lipoprotein (HDL-C), and other blood lipid parameters were all measured.

### Doppler Echocardiography

Determining left ventricular functional parameters: Doppler echocardiography was performed by color ultrasound, and left ventricular ejection fraction (LVEF) was obtained by M-mode echocardiography and apical four-chamber view. The following parameters were measured: early diastolic peak velocity (E), late diastolic peak velocity (A), and early diastolic mitral annulus velocity (e’). And the values of E/A were calculated as the ultrasonic diagnostic criteria of diastolic cardiac dysfunction, while the values of E/e’ were used to assess systolic and diastolic myocardium and for the estimation of left ventricular filling pressure.

### Serum Sample Preparation for Metabolic Profiling

The samples were thawed on ice before three volumes of ice-cold methanol were added to 1 volume of plasma/serum, which was then whirled for 2 min and incubated at −20°C for 0.5 h. The mixture was then whirled for 2 min before being centrifuged at 12,000 rpm for 10 min at 4°C. The supernatant was collected and incubated for 0.5 h at −20°C. Finally, it was centrifuged for 15 min at 12,000 rpm at 4°C, and the supernatant was collected for LC-MS/MS analysis.

### UPLC-MS/MS Analysis

The analytical conditions of UPLC-MS/MS were as follows: UPLC column, Waters ACQUITY UPLC HSS T3 C18 (1.8 µm, 2.1 mm × 100 mm); column temperature, 40°C; flow rate, 0.4 ml/min; injection volume, 2 μL; solvent system, water (0.1% formic acid): acetonitrile (0.1% formic acid); gradient program, 95:5 V/V at 0 min, 10:90 V/V at 11.0 min, 10:90 V/V at 12.0 min, 95:5 V/V at 12.1 min, 95 : 5 V/V at 14.0 min.

### Multivariate Data Processing and Data Analysis

The original data file obtained by UPLC-MS analysis was firstly converted into mzML format by ProteoWizard software. Peak extraction, alignment, and retention time correction were performed by the XCMS program. The “SVR” method was used to correct the peak area. Peaks with deletion rate >50% in each group of samples were filtered out. After that, metabolic identification information was obtained by searching the laboratory’s self-built database and integrating the public database and metDNA. Finally, a statistical analysis was carried out by the R package. The statistical analysis included univariate analyses and multivariate analyses. Univariate statistical analyses included the Student’s t-test and variance multiple analysis. Multivariate statistical analyses included principal component analysis (PCA), partial least squares discriminant analysis (PLS-DA), and orthogonal partial least squares discriminant analysis (OPLS-DA). All experimental data were presented as the mean ± standard error. *p* < 0.05 was considered statistically significant.

## Results

### Research Population for Non-Targeted Metabonomic Analysis

A total of 78 patients with T2DM were sampled in this study, including group I (DCM group) containing 39 type 2 diabetes patients with myocardial diastolic dysfunction and group II (DM group), including 39 type 2 diabetes patients without myocardial diastolic dysfunction. No significant difference in clinical characteristics was observed between the two groups, as shown in Table 1. The non-targeted metabonomic screening data set was obtained from 78 patients with mass spectrometric analysis.

**TABLE 1:** Description of the sample population in this study.

Clinical parameters	DM (*n* = 39)	DCM (*n* = 39)	*p* Value
BMI	24.19 ± 0.44	23.77 ± 0.40	0.48
WHR	0.93 ± 0.01	0.94 ± 0.01	0.49
HbA1c	9.25 ± 0.41	8.39 ± 0.31	0.10
FBG	7.50 ± 0.54	6.93 ± 0.37	0.39
PBG	14.96 ± 0.85	16.11 ± 0.87	0.35
TG	1.71 ± 0.29	1.33 ± 0.15	0.25
TC	4.56 ± 0.23	4.43 ± 0.18	0.66
LDL	2.80 ± 0.17	2.76 ± 0.16	0.87
HDL	1.09 ± 0.07	1.14 ± 0.03	0.51

BMI, body mass index; HbA1c, glycated hemoglobin; WHR, waist-to-hip ratio; FBG, fasting blood glucose; TG, triglyceride; TC, total cholesterol; LDL, low-density lipoprotein cholesterol; HDL, high-density lipoprotein cholesterol.

### Heart Function Assessment With Doppler Echocardiography

Conventional echocardiographic parameters related to LV structure and function are described in Table 2. The E (70.59 ± 3.01 vs. 84.10 ± 2.59, *p* < 0.01) and e’ (6.61 ± 0.23 vs. 9.40 ± 0.38, *p* < 0.001), represent the maximum blood flow in the early diastolic left ventricle and are significantly reduced in the DCM group compared to that of the DM group. The A-peak, which reflects left atrial systolic hemodynamics, was significantly elevated in the DCM group compared to that of the DM group (86.79 ± 3.24 vs. 68.21 ± 2.80, *p* < 0.001). As a result, the E/A and E/e’ ratios in the DCM were significantly lower than that in the DM group. There was no significant difference between the two groups regarding resting LV dimension, LV mass index, and LV ejection fraction.

**TABLE 2:** Description of the heart function of the patients in two groups.

	DM	DCM
E	84.10 ± 2.59	70.59 ± 3.01**
e’	9.40 ± 0.38	6.61 ± 0.23***
A	68.21 ± 2.80	86.79 ± 3.24***
LVEF	67.51 ± 0.76	66.05 ± 0.84
E/A	1.29 ± 0.05	0.83 ± 0.03**
E/e’	9.28 ± 0.43	11.10 ± 0.60***
FS	37.56 ± 0.62	36.36 ± 0.64
ROVT	26.64 ± 0.65	27.62 ± 0.68
AO	28.38 ± 0.48	29.33 ± 0.54
LA	30.59 ± 0.56	31.42 ± 0.60
IVS	9.86 ± 0.22	9.77 ± 0.19
LVPW	9.40 ± 0.16	9.28 ± 0.20
LVD	45.97 ± 0.52	45.62 ± 0.68
LVS	28.49 ± 0.45	29.03 ± 0.56
PA	21.23 ± 0.28	21.59 ± 0.21
V-PA	89.92 ± 2.81	88.61 ± 2.07
V-LOVT	106.97 ±2.83	104.56 ± 2.82

**p < 0.01 vs DM; ***p < 0.001 vs DCM.

### Orthogonal Partial Least Square Discriminant Analysis (OPLS-DA)

**FIGURE 1:**
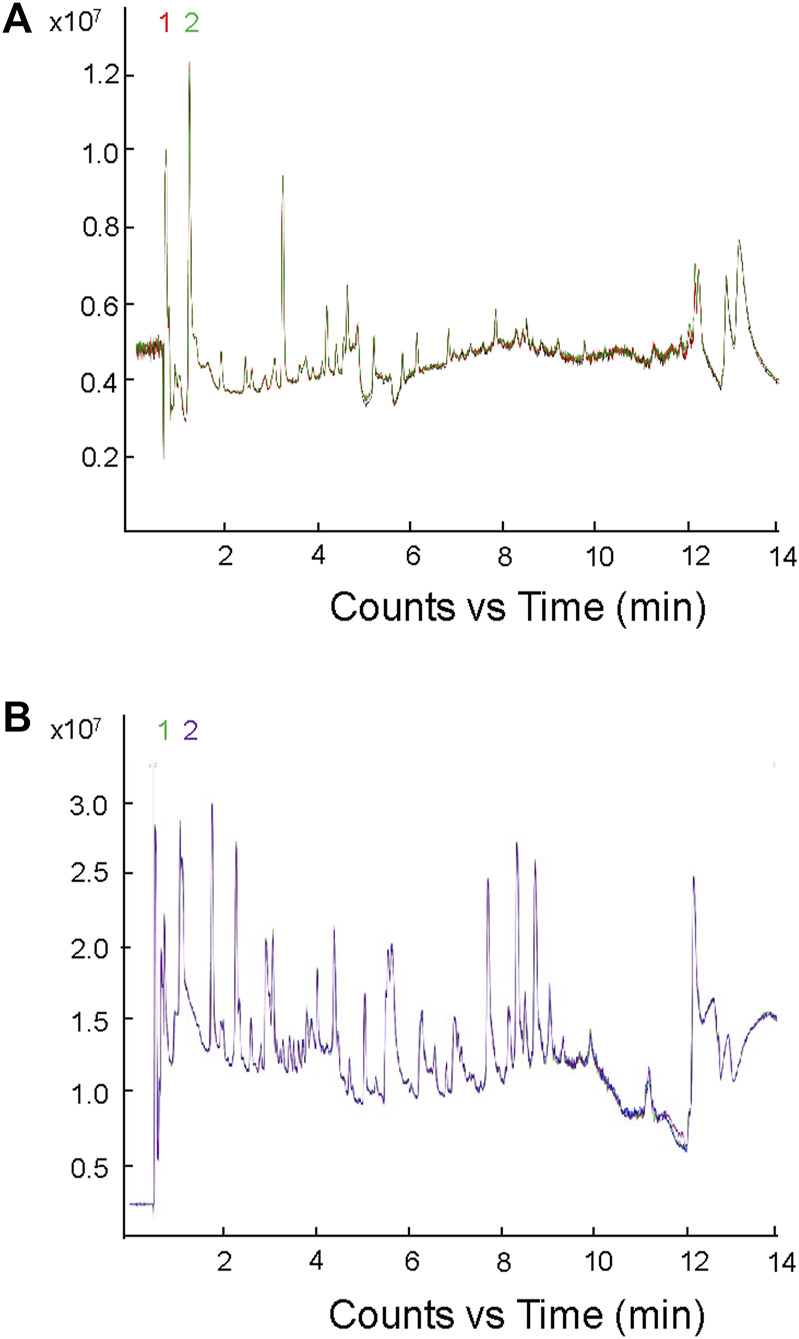
Mass spectrometric analysis of TIC overlap map of mixed samples. **(A)** Detection of TIC overlap map of QC samples in positive ion mode; **(B)** detection of TIC overlap map of QC samples in negative ion mode.

The metabolome group data were subjected to data quality control (QC) analysis. As shown in Figure 1, all the QC sample’s peaks overlapped well, and there was little difference in peak intensity fluctuation, indicating that the data were reliable and qualified for the subsequent analysis.

**FIGURE 2:**
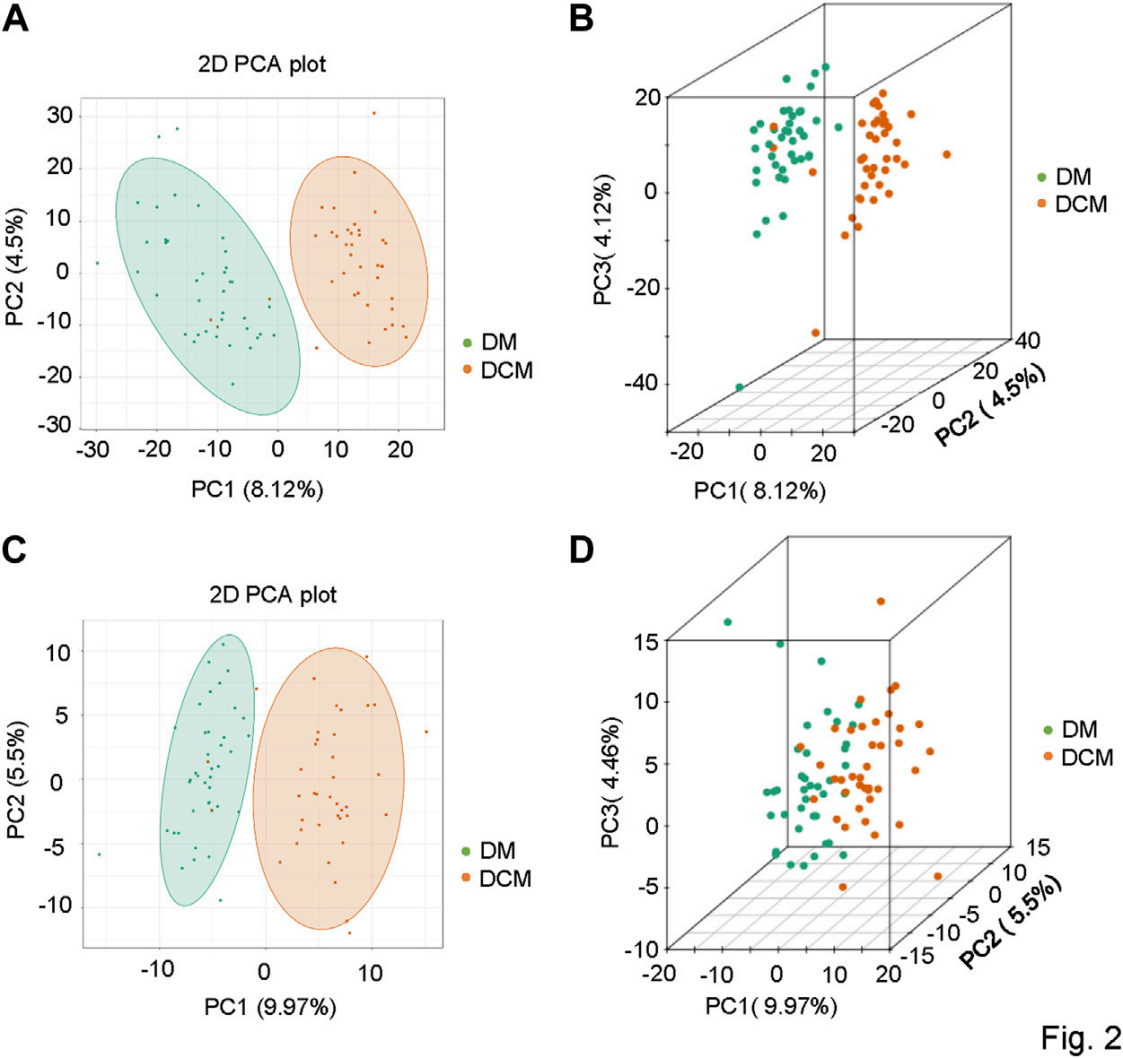
Principal component analysis diagrams. **(A)** PCA diagram of corresponding groups in the positive ion mode; **(B)** three-dimensional diagram of corresponding groups in the positive ion mode; **(C)** corresponding grouping PCA diagram in the negative ion mode; **(D)** three-dimensional diagram of corresponding grouping PCA in the negative ion mode.

To understand the general differences of metabolites in all samples, the principal component analysis (PCA) on the abundances of metabolites was conducted. A clear trend of partial separation of metabolites between groups was observed in PCA. The PCA score in positive and negative ion modes was shown as follows: the PC1s of DCM and DM in positive and negative ion modes were 8.12 and 9.97%, respectively (Figure 2), indicating metabolic differences between these two patient groups.

**FIGURE 3:**
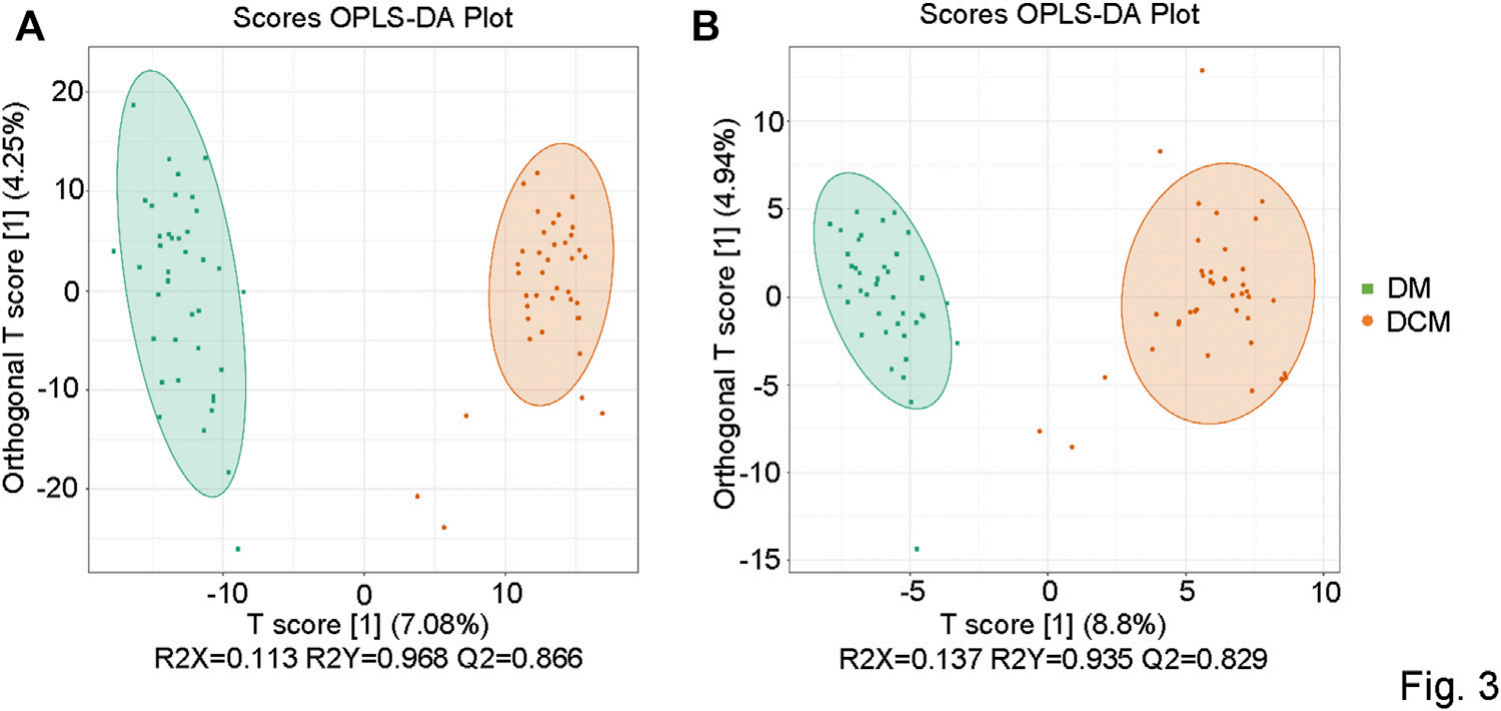
OPLS-DA score chart. **(A)** OPLSDA score diagram of corresponding groups in the positive ion mode; **(B)** OPLSDA score diagram of corresponding groups in the negative ion mode.

The metabolic datasets were further analyzed with the orthogonal partial least squares’ discriminant analysis (OPLS-DA) model. The components of independent variable X and dependent variable Y were extracted, respectively, and the correlation between the components was calculated. R2X and OPLS-DA score maps were used to evaluate the classification effect of the model, and the score maps of OPLS-DA groups were drawn (Figure 3). The closer the three indexes were to 1, the more stable and reliable the model was. For the metabolites from the positive ion mode, the T-score was 7.08%, and the Magi Orthogonal T-score was 4.25%. For the metabolites from the negative-ion mode, the T-score was 8.8%, and the Magi Orthogonal T-score was 4.94%. The OPLS-DA score map reflected the better data separation between DCM and DM in the positive and negative ion mode. There was a significant difference between the two groups, which confirmed the reliability of our model.

**FIGURE 4:**
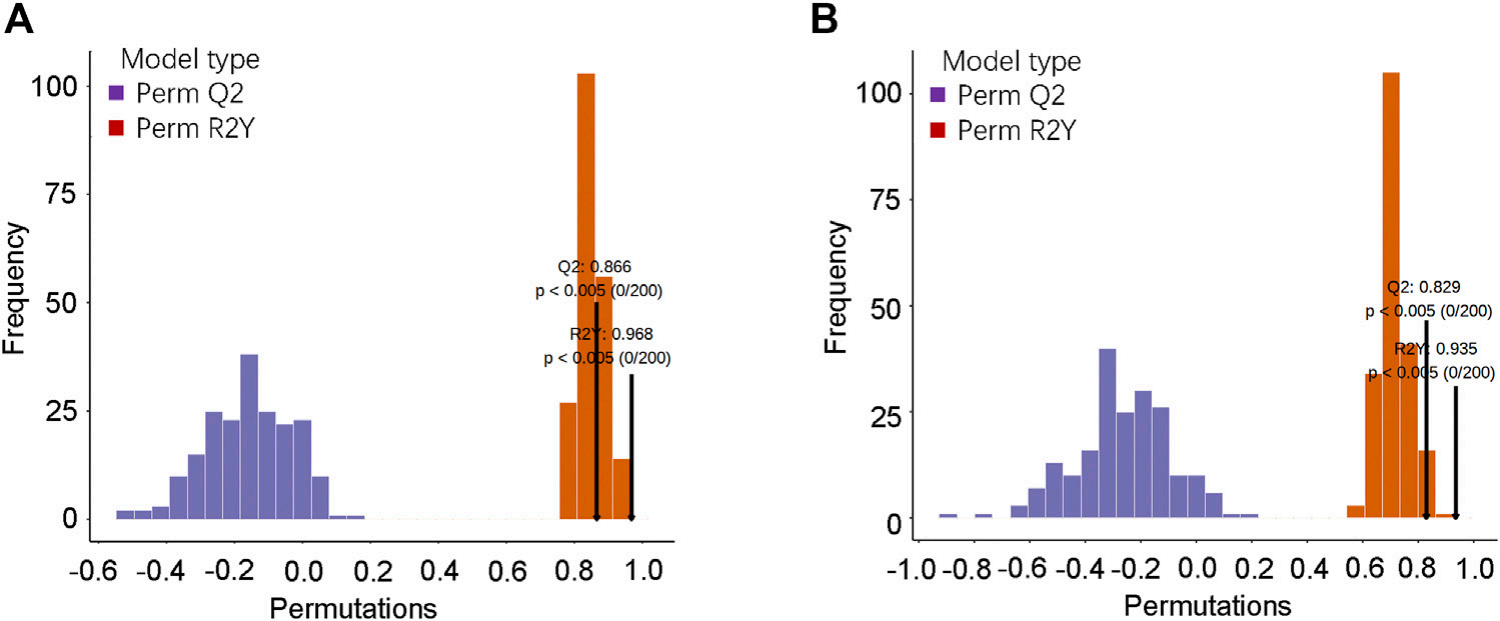
OPLS-DA model verification. **(A)** Verification diagram of the corresponding grouping OPLSDA model in the positive ion mode; **(B)** verification diagram of the corresponding grouping OPLSDA model in the negative ion mode.

To further verify the OPLS-DA model, a verification Permutation test analysis was conducted (Figure 4). In positive ion mode, Q2 0.866 indicated that the prediction ability of 173 random grouping models in this permutation detection was better than that of the OPLS-DA model, and R2Y was 0.968, indicating that the interpretation rate of the Y matrix of 193 random grouping models in this permutation detection was better than that of the OPLS-DA model. In the negative ion mode, Q2 0.829 indicated that the prediction ability of 165 random grouping models in this permutation detection was better than that of the OPLS-DA model; R2Y was 0.935, indicating that the explanation rate of the Y matrix of 187 random grouping models in this permutation detection was better than that of the OPLS-DA model. In both ion modes, *p*-values < 0.05 was statistically significant. The abovementioned results verified the effectiveness of the model.

## Differential Metabolite Screening

**FIGURE 5:**
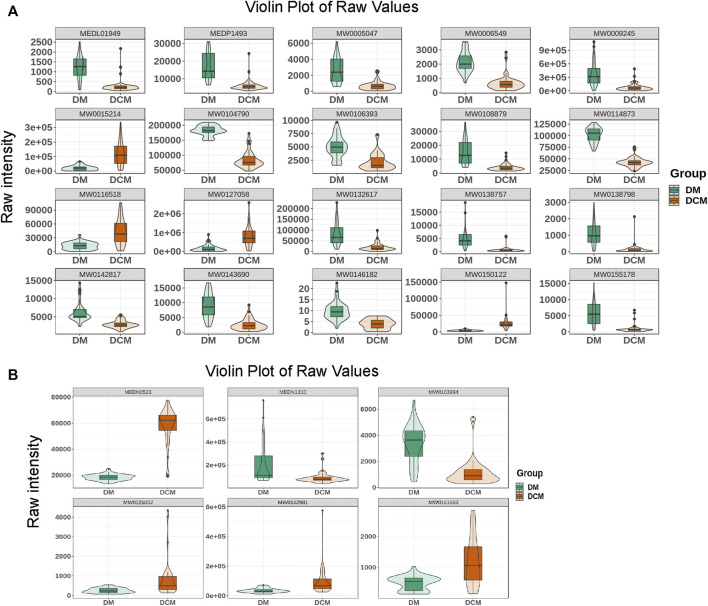
Violin diagram. **(A)** The violin diagram of the top known differential metabolites in the positive ion mode; **(B)** the violin diagram of the top known differential metabolites in the negative ion mode.

Based on the OPLS-DA results, the metabolites of different groups could be preliminarily screened from the variable importance projection (VIP) of the multivariate analysis OPLS-DA model. The VIP value indicates the influence intensity of the difference of the corresponding metabolites between groups, and it is generally believed that the difference of the metabolites with VIP ≥1 is significant. Here, the differential metabolites were screened by combining the *p*-value of univariate analysis, the multiple of difference (fold change), and the VIP value of the OPLS-DA model. The log FC ≥ 2, VIP ≥1 and *p*-value < 0.05 were classified as differential metabolite. According to this criterion, there were 78 differential metabolites in the positive ion mode, including 33 upregulated and 45 downregulated metabolites (shown in Supplementary Table 3). However, there were only 6 differential metabolites identified in the negative mode, including 4 up-regulated and 2 down-regulated differential metabolites. Differential metabolites include terpenoids, phenylpropanoids and polyketides, vitamins, organoheterocyclic compounds, organic oxygen compounds, organic nitrogen compounds, organic acids and derivatives, nucleotide and its metabolomics, lipids and lipid-like molecules, heterocyclic compounds, carboxylic acids and derivatives, benzenoids, amino acids and derivatives, alkaloids and derivatives, and alcohol and amines (Figure 5).

## Analysis of Differential Metabolites

**FIGURE 6:**
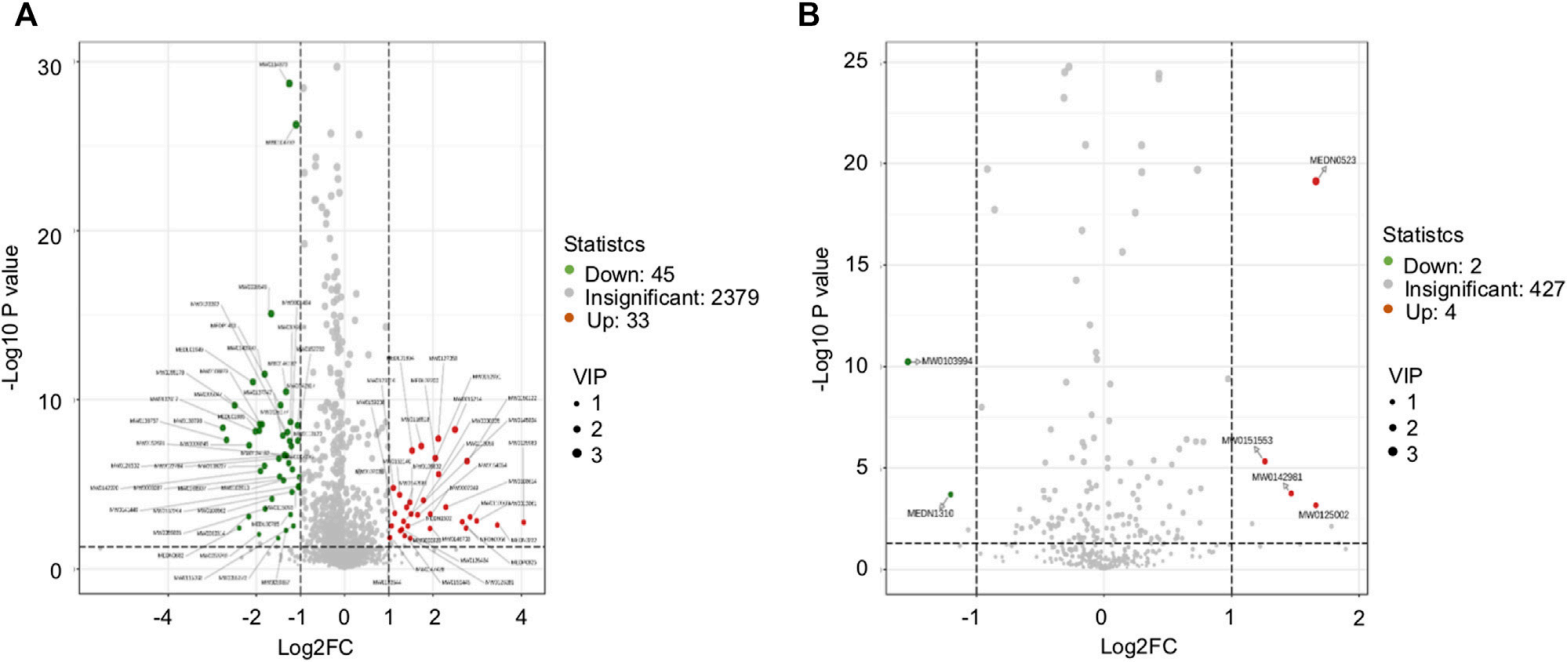
Volcanic map. **(A)** Volcanic diagram of the corresponding grouping under positive ion mode; **(B)** volcanic diagram of the corresponding grouping under negative ion mode; the red for the significant upregulation of the metabolite, the green for the significant (*p* < 0.05) downregulation of the metabolite, and the gray for the non-significant metabolite.

The difference in the abundance level of metabolites between the DCM and DM group was examined and plotted on a volcanic map (Figure 6), where a statistically significant cutoff was at *p* < 0.05. In the positive ion mode, 33 up-regulated differentially expressed metabolites, and 45 down-regulated differentially expressed metabolites were obtained. In the negative ion mode, four up-regulated differentially expressed metabolites, and two down-regulated differentially expressed metabolites were obtained (Table 3).

**TABLE 3:** Identification results of the top 20 differential regulated metabolites.

Index	Compounds	Log_2_FC	VIP	Regulation
MW0104790	2-Imino-1-imidazolidineacetic acid	−1.1	3.51	Down
MW0114873	Metyrapone	−1.26	3.45	Down
MW0006549	Chlorothalonil	−1.67	2.92	Down
MW0143690	5-Oxo-d-bilirubin	−1.81	2.66	Down
MW0146182	Asp Lys Arg Glu Lys	−1.33	2.63	Down
MEDL01949	Gossypol	−2.08	2.6	Down
MW0155178	Phe Glu His Asp	−2.49	2.52	Down
MW0015214	7-Keto-8-aminopelargonic acid	2.5	2.48	Up
MEDP1493	N6-methyladenosine	−1.45	2.48	Down
MW0132617	3′,4′-Methylenedioxyorobol	−2.02	2.44	Down
MW0108879	N-omega-Hydroxy-L-arginine	−1.87	2.38	Down
MW0005047	4-Hydroxy-3-(3-methylbut-2-en-1-yl)benzoic acid	−1.92	2.37	Down
MW0138798	Linarin	−2.76	2.37	Down
MW0116518	1-(Piperidin-2-yl)propan-1-one	1.74	2.35	Up
MW0009245	O-Desmethylmycophenolic acid	−2.16	2.33	Down
MW0142817	3b-(1-Pyrrolidinyl)-5α-pregnane-11,20-dione	−1.07	2.32	Down
MW0127058	δ-Undecalactone	2.12	2.32	Up
MW0150122	Glu Pro Gly Tyr Ser	2.77	2.31	Up
MW0138757	Leucoside	−2.68	2.3	Down
MW0106393	D-Glutamine	−1.23	2.29	Down
MEDN0523	Indole-3-lactic acid	1.66	3.12	Up
MW0103994	(S)-2-Hydroxyglutaric acid	−1.54	2.33	Down
MW0151553	Ile Phe Val Lys	1.26	1.73	Up
MW0142981	3-Hydroxyethylchlorophyllide a; 3-devinyl-3-(1-hydroxyethyl)chlorophyllide a	1.47	1.5	Up
MEDN1310	5-Hydroxymethyl-2-furancarboxylic acid	−1.2	1.48	Down
MW0125002	Methohexital	1.66	1.33	Up

VIP for variable importance in projection, log2FC for log2 transformation of folder change.

The top 20 differential metabolites with the highest VIP value were selected to draw the violin map (Figure 5) and listed in Table 4.

**FIGURE 7:**
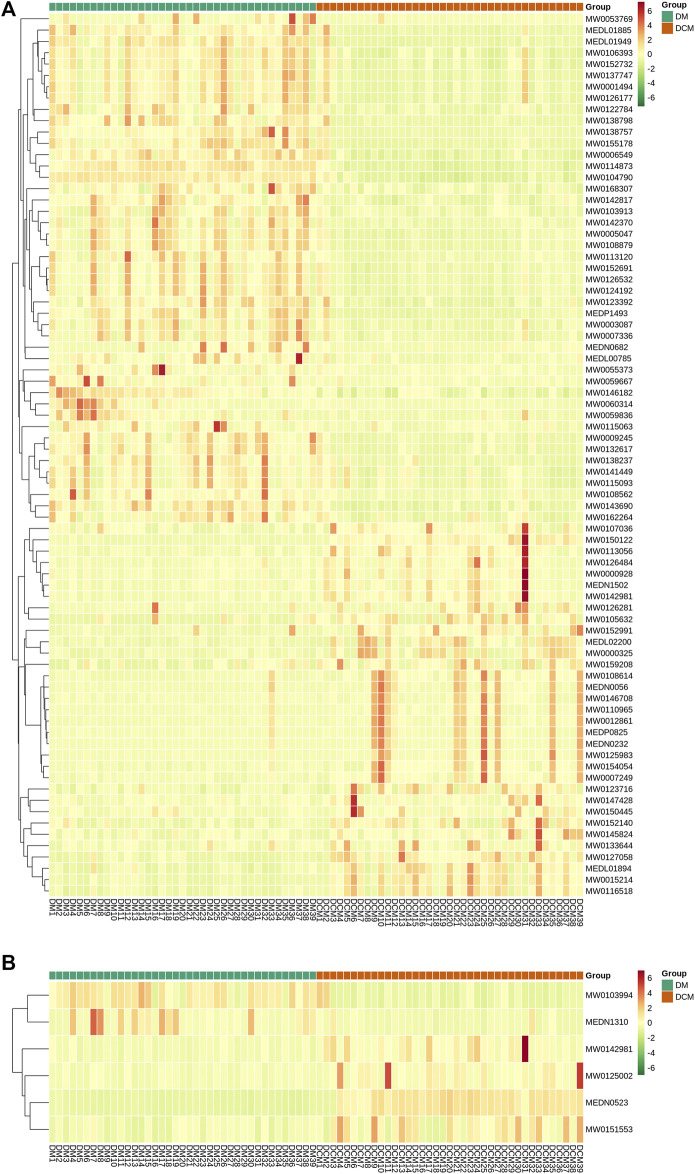
Heat maps of differential metabolites from serum. Rows: metabolites; columns: samples. **(A)** Cluster analysis heat map of differential metabolites known in corresponding groups in positive ion mode; **(B)** cluster analysis heat map of differential metabolites known in related groups in negative ion mode.

Cluster analysis was conducted for differential metabolites and the significant changes of these metabolites were visualized with heat maps (Figure 7). Based on the cluster analysis of the differential metabolites in positive and negative ion modes, the differential metabolites in DM and DCM patients were utterly divided into two categories. Most of the metabolites were increased in all serum samples of DCM patients.

**FIGURE 8:**
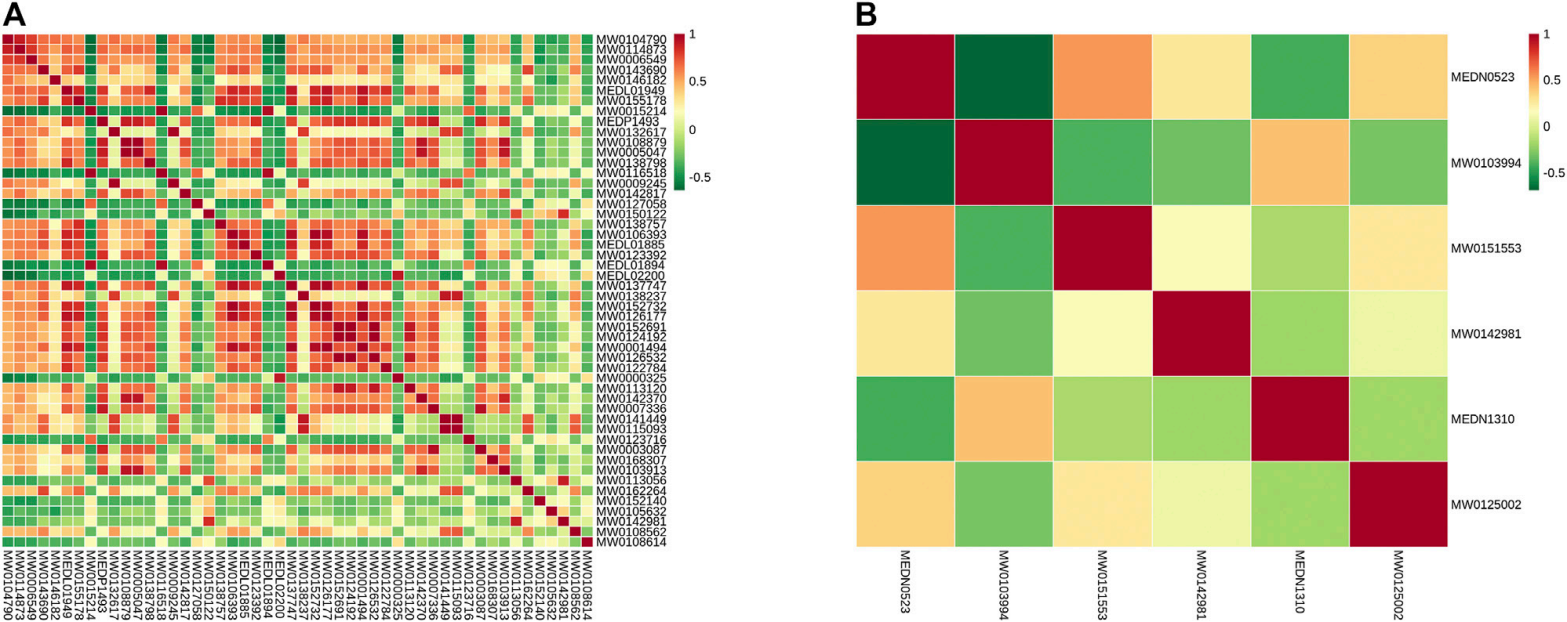
Differential metabolite correlation map. **(A)** Grouping in the positive ion mode; **(B)** grouping in the negative ion mode; red indicates strong positive correlation, and green indicates strong negative correlation.

According to the correlation analysis of differential metabolites, the significant difference in metabolites was analyzed by the Pearson correlation coefficient (PCC). The differential metabolite correlation heat map was drawn (Figure 8). In the positive ion mode, there was a high correlation between many differential metabolites. While in the negative ion mode, the correlation between the differential metabolites was not significant. This difference may be due to the small number of differential metabolites in the negative ion mode.

**FIGURE 9:**
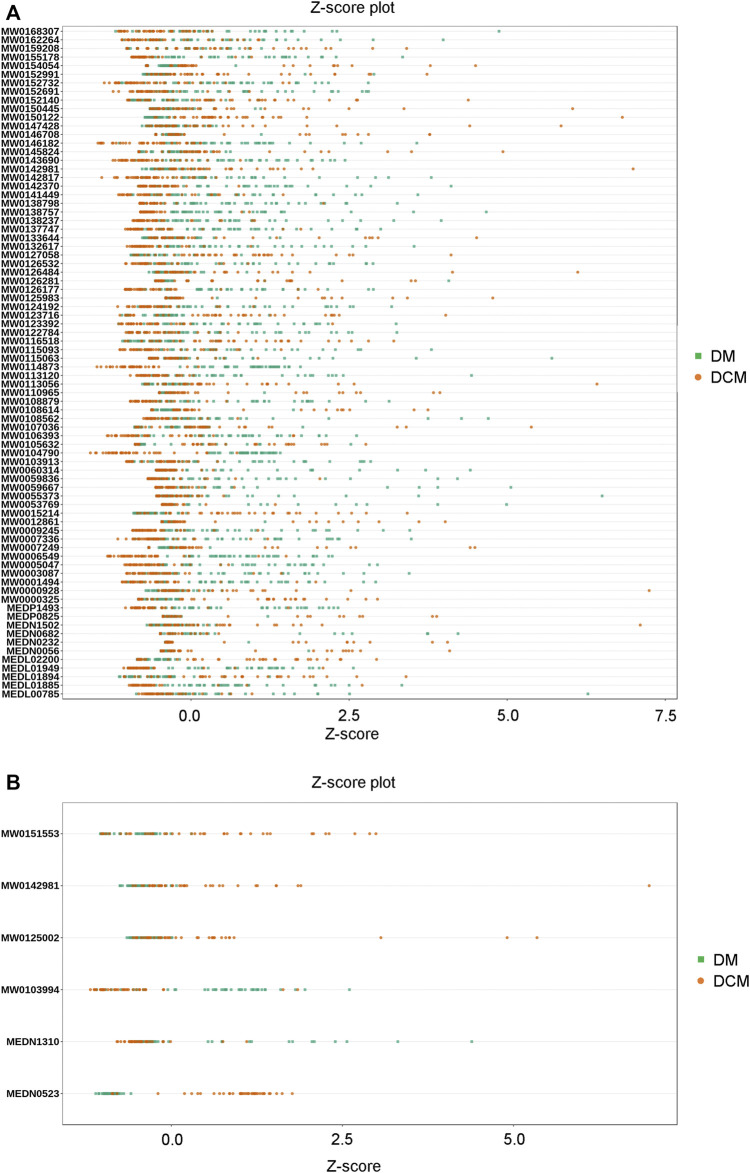
Z-value map of differential metabolite. **(A)** Z-value diagram of the known differential metabolites in corresponding groups in the positive ion mode; **(B)** Z-value diagram of known differential metabolites in corresponding groups in the negative ion mode; different colors of the points represent different groups of samples DM or DCM.

The Z-score analysis was used to normalize the differential metabolites in different samples (Figure 9). The visualization of the distribution showed that in the positive ion mode, the expression of differential metabolites in serum samples of DCM patients was relatively stable, while the expression of differential metabolites in serum samples of DM patients varied greatly; In the negative ion mode, the levels of differential metabolites in serum samples of both DCM and DM patients were significantly changed.

## KEGG Enrichment Analysis of Differential Metabolites

**FIGURE 10:**
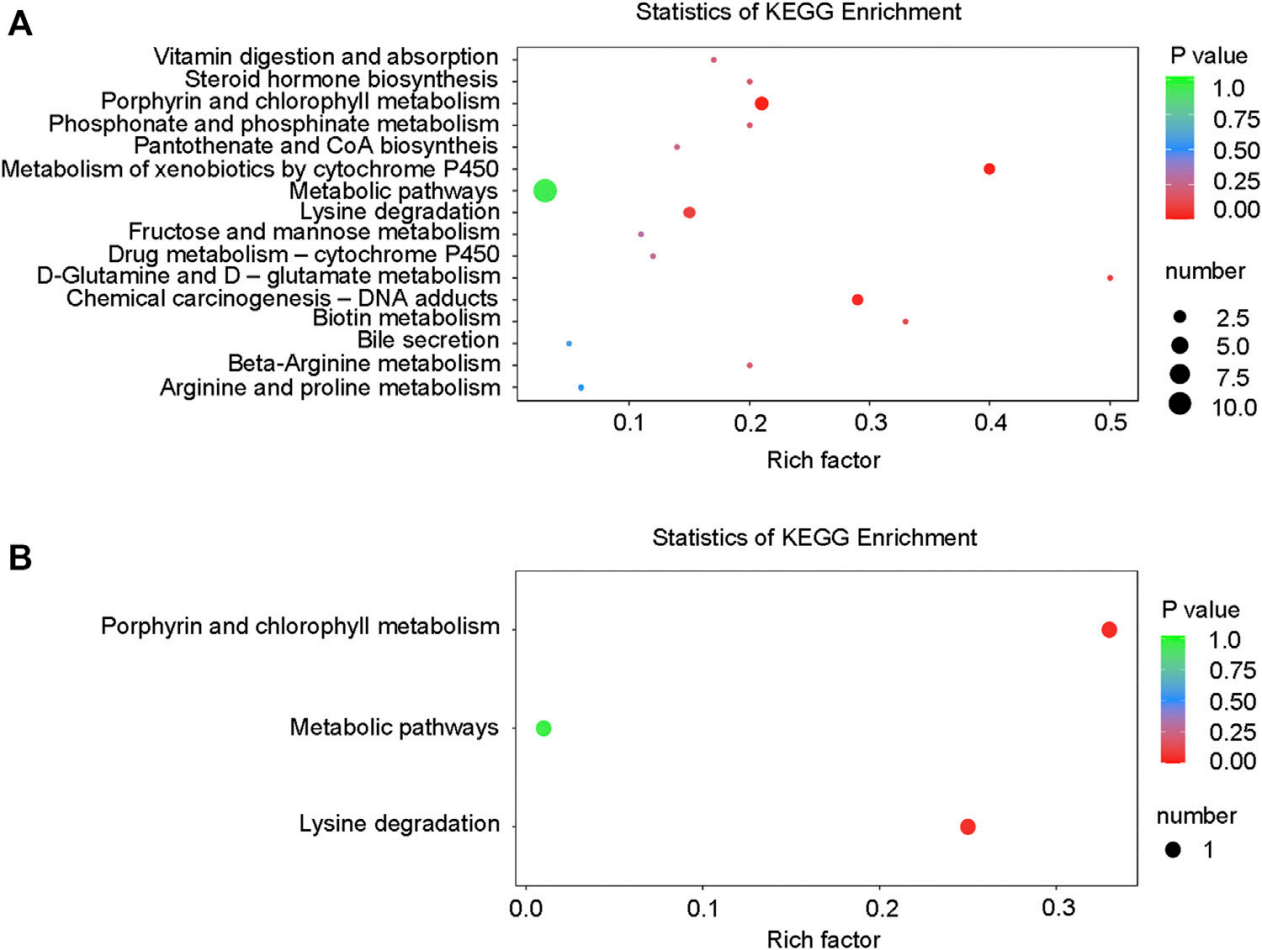
KEGG enrichment map of differential metabolites. **(A)** KEGG differential enrichment bubble diagram in the positive ion mode; **(B)** KEGG differential enrichment bubble diagram in the negative ion mode.

Differential metabolites interact with each other to form different pathways in organisms. The differential metabolites were annotated and displayed with KEGG (Kyoto Encyclopedia of Genes and Genomes). KEGG pathway enrichment of differential metabolites was carried out (Figure 10), and the results were as follows: in the positive ion mode, the differential metabolites were significantly enriched in 16 pathways (Table 4), while in the negative ion mode; the differential metabolites were significantly enriched in 3 pathways (Table 4). These signaling pathways included porphyrin and chlorophyll metabolism, metabolism of xenobiotics by cytochrome P450, chemical carcinogenesis, D-Glutamine, and D-glutamate metabolism, lysine degradation, biotin metabolism, β-alanine metabolism, phosphonate and phosphinate metabolism, steroid hormone biosynthesis, vitamin digestion, absorption, pantothenate and CoA biosynthesis, drug metabolism-cytochrome P450, fructose and mannose metabolism, arginine, proline metabolism, and bile secretion (Figure 10). These pathways were related to glucose and lipid metabolism, amino acid metabolism, oxidative energy supply, and inflammation, which were closely related to the pathogenesis of DCM.

**TABLE 4:** KEGG pathways involved by differentially regulated metabolites.

Pathway(POS)	ID	Unique compound	Compound	*p*-value
Steroid hormone biosynthesis	ko00140	1	5	0.190
Metabolic pathways	ko01100	11	323	0.947
Metabolism of xenobiotics by cytochrome P450	ko00980	2	5	0.015
Chemical carcinogenesis	ko05204	2	7	0.030
b-Alanine metabolism	ko00410	1	5	0.190
Pantothenate and CoA biosynthesis	ko00770	1	7	0.257
Vitamin digestion and absorption	ko04977	1	6	0.224
Lysine degradation	ko00310	2	13	0.095
Porphyrin and chlorophyll metabolism	ko00860	3	14	0.016
Phosphonate and phosphinate metabolism	ko00440	1	5	0.190
Fructose and mannose metabolism	ko00051	1	9	0.318
D-Glutamine and D-glutamate metabolism	ko00471	1	2	0.080
Biotin metabolism	ko00780	1	3	0.119
Drug metabolism—cytochrome P450	ko00982	1	8	0.288
Bile secretion	ko04976	1	21	0.596
Arginine and proline metabolism	ko00330	1	18	0.538
Pathway(NEG)	ID	Unique compound	Compound	*p*-value
Lysine degradation	ko00310	1	4	0.048
Metabolic pathways	ko01100	1	130	0.956
Porphyrin and chlorophyll metabolism	ko00860	1	3	0.036

## Discussion

**FIGURE 11:**
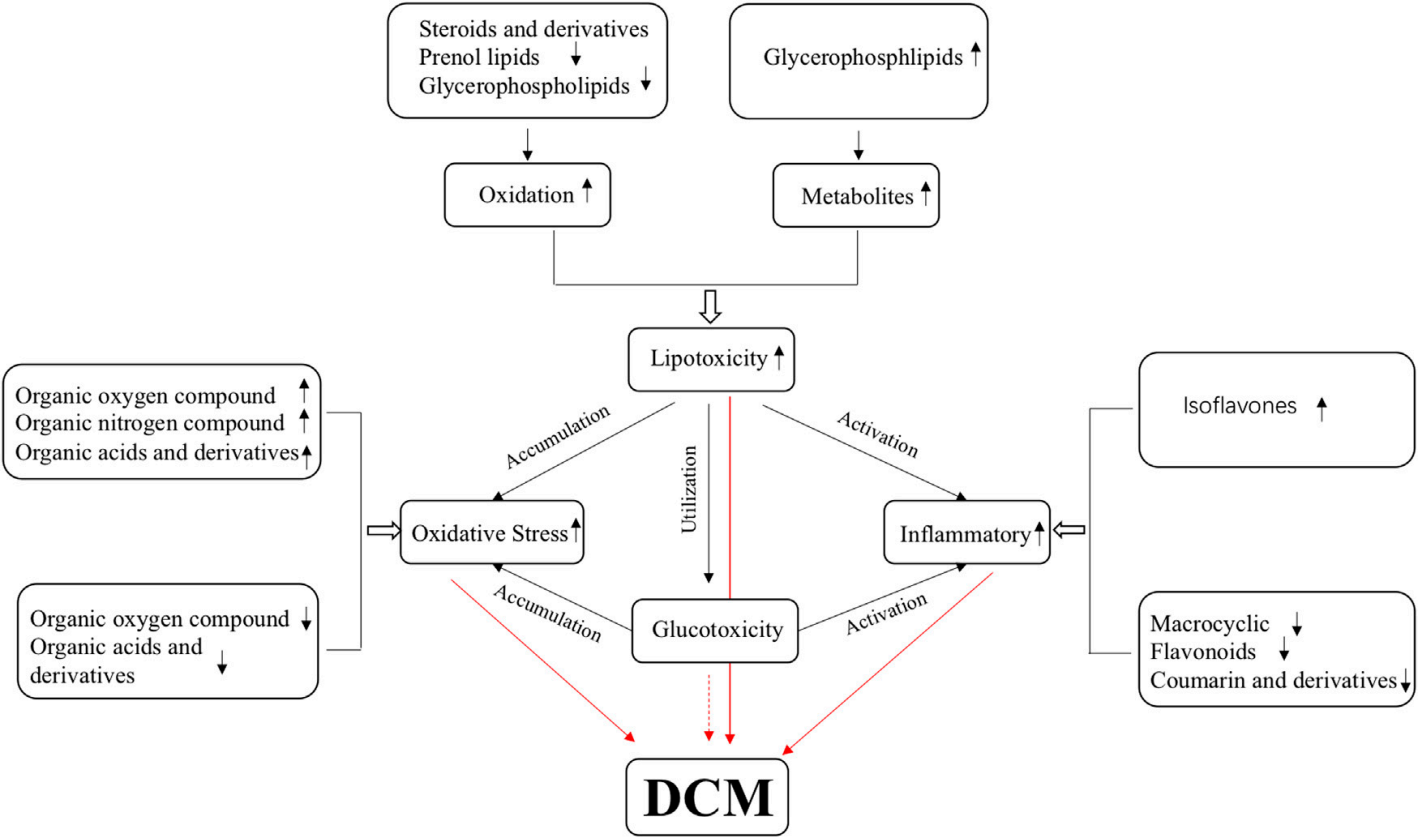
Summary of the possible metabolites and metabolomic pathways in diabetic cardiomyopathy.

Diabetic cardiomyopathy is the main complication of diabetes and the leading cause of death in patients with diabetes (Dandamudi et al., 2014). Myocardial diastolic dysfunction occurs in the early stages of DCM and is easily ignored, so the early cognition of DCM diastolic dysfunction is essential. In this study, differential metabolites in serum were identified between DCM and DM, and significantly enriched in metabolism pathways for 37 up-regulated differential metabolites and 47 down-regulated differential metabolites in both ion modes. Of those differential metabolites, terpenoids have been reported to have an anti-inflammatory effect and can regulate blood lipid and cardiotonic activity (Barsby et al., 1993; Kim et al., 2020). Here their expression in DCM was downregulated. Phenylpropanoids and polyketides have anti-inflammatory effects, and most of these metabolites are downregulated in DCM (Fiorito et al., 2019). Organoheterocyclic compounds, organic oxygen compounds, and organic nitrogen compounds are involved in a redox reaction and these metabolites were also changed in DCM. Lipids and lipid-like molecules affect the glucose and lipid metabolism of DCM, and most of these metabolites are downregulated in DCM (Cohain et al., 2021). These differential metabolites are closely related to the pathogenesis, occurrence, and development of DCM. Thus, these differential metabolites should be potentially valuable biomarkers to guide clinical diagnosis and treatment in the pathophysiological process of DCM (Figure 11).

The disorder of cardiac glucose and lipid metabolism and the imbalance of energy supply plays an essential role in the occurrence and development of DCM. Excess-free fatty acids will be released in diabetic patients, resulting in lipid toxicity in cardiomyocytes (Jia et al., 2016; Jia et al., 2018). Excessive intracellular lipid metabolic intermediates, such as diacylglycerol and ceramide, will promote the damage of insulin metabolic signal, apoptosis, and fibrosis of cardiomyocytes and aggravate the occurrence and development of DCM (Sharma et al., 2008; Li et al., 2020). A recent study suggests that these alterations in cardiac energy metabolism precede the development of glucose intolerance and cardiac hypertrophy and that therapeutically modulating cardiac energy metabolism by reducing fatty acid oxidation and increasing glucose oxidation may improve cardiac function in DCM (Buchanan et al., 2005). This study found that some lipid-associated compounds accumulated while some were down-regulated, including steroids and steroid derivatives, prenol lipids, and glycerol phospholipids. The eight down-regulated lipids and lipid metabolites may be used for energy oxidation, and one up-regulated lipid metabolite may be the accumulated intermediates. Our results further confirmed that the ability of the myocardium to utilize glucose is declined, and its energy supply may mainly be dependent on fatty acid oxidation.

Growing evidence points to the potential involvement of oxidative stress in the pathophysiology of DCM. Abnormal lipid metabolism (Qi et al., 2013) and mitochondrial dysfunction (Pal et al., 2017) can stimulate cardiomyocytes to produce ROS and induce oxidative stresses. The accumulation of ROS can induce the formation of advanced glycation end products (advanced glycation end products, AGEs), stimulate the expression of AGEs receptor, and inhibit the activity of NO synthase (nitric oxide synthase, NOS) and prostacyclin synthase (prostacyclin synthetase, PGIS), which will promote myocardial fibrosis, diastolic dysfunction and even heart failure in patients with diabetes (Quan et al., 2020). This study obtained four down-regulated organic oxygen compounds, one up-regulated organic oxygen compound and one up-regulated organic nitrogen compound, eight down-regulated organic acids and derivatives, and seven up-regulated organic acids and derivatives in DCM. This is consistent with previous findings (Yin et al., 2019), showing that the ROS is also a good candidate marker.

Inflammatory factors are involved in DCM development leading to cardiac remodeling, fibrosis, and diastolic dysfunction (Li et al., 2020). Hyperglycemia and high free fatty acid levels also activate NLRP3 inflammatory bodies that activate reactive oxygen species pathways (Deery et al., 2012). In diabetic heart tissue, the polarization of pro-inflammatory macrophages M1 is upregulated, while the anti-inflammatory response of macrophage M2 is inhibited (Hansen et al., 2017). This study obtained five down-regulated phenylpropane and polyketone compounds, including macrocyclic lactam, coumarin and derivatives, flavonoids, and up-regulated phenylpropane and polyketone isoflavones. A series of studies reported that phenylpropane and polyketone compounds have significant anti-inflammatory and antioxidant effects (Wang et al., 2012; Gasparrini et al., 2016; Wang et al., 2016; Ju et al., 2017). Their downregulation in DCM serum indicates that the anti-inflammatory and antioxidant effects are weakened in DCM patients, while oxidation and inflammation occur in DCM.

Many pathways are closely related to the pathogenesis of DCM. The identified 19 pathways in our results were associated with many aspects of metabolisms, including glucose, energy, lipid, amino acid, inflammation, and other biological processes. These pathways contain 18 kinds of unique compounds, including 12 kinds of unique compounds in the metabolic pathway. They are five kinds of organic acids and derivatives, one lipid and lipid-like molecule, which are steroids and steroid derivatives, one kind of pantothenate, chlorophyllide, and four kinds of amino acids. They jointly affect the function of metabolic pathways. Our findings are consistent with previous works. Chlorophyllin alleviates hyperglycemia-induced oxidative stress and apoptosis in the liver of streptozotocin-administered mice (Patar et al., 2018). The enrichment of the rich factor in the lysine degradation pathway is significant and reliable, including three kinds of unique compounds, all of which are organic acids and derivatives. The porphyrin and chlorophyll metabolism pathway contains four types of unique compounds, all of which are organoheterocyclic compounds. Among them, the porphyrin and chlorophyll metabolism signal pathway is involved in vitamin B12 metabolism (Bolton et al., 2000), while vitamin B12 deficiency is related to cardiovascular autonomic neuropathy in patients with type 2 diabetes mellitus (Hansen et al., 2017). Metabolism of xenobiotics by cytochrome P450 pathways rich factor enrichment is significant and reliable, including two unique compounds, organoheterocyclic compounds, and organooxygen compounds. The study provides evidence that diabetes initiates cardiomyopathy by increasing sEH, reducing cytochrome P450 2J, and decreasing cardioprotective EETs, finally attenuating cardiotoxicity mediated by the reduction of high glucose in cardiac cells (Alaeddine et al., 2021). In addition, cytochrome P450 has a close relationship with inflammation in T2DM thought to decrease P450 isoenzymes and rise in plasma levels of these enzymes, finally resulting in high expression of interleukin-6 (IL-6) and the tumor necrosis factor-alpha (TNF-α) (Darakjian et al., 2021). These results above confirm that the signaling pathway of cytochrome P450 plays a vital role in diabetic cardiomyopathy, and our results provide another evidence. In addition, some previously known metabolisms were also identified in this study, for example, D-glutamine and D-glutamate metabolism pathway, biotin metabolism pathway, chemical carcinogenesis pathway, rich factor enrichment is significant and reliable, which suggests the common and key pathways for some diseases.

To summarize, this study provides important clues for the study of the regulation of metabolites and metabolites pathways in DCM. In future research, we still need more studies to verify their regulation and narrow down the key metabolite candidates in the relevant cell experiments and animal models, and explore the mechanism of metabolites affecting DCM.

## Data Availability

The original contributions presented in the study are included in the article/Supplementary Material, further inquiries can be directed to the corresponding authors.
