# Supplementary material for: Metabonomic Characteristics of Myocardial Diastolic Dysfunction in Type 2 Diabetic Cardiomyopathy Patients
Source: Front Physiol. 2022 May 9;13:863347. doi: 10.3389/fphys.2022.863347 (PMC9150260; doi:10.3389/fphys.2022.863347)
Supplement: Supplementary file 2 [file Table1.pdf]

Supplement table-1.Chromatographic column mobile phase gradient condition T3

**Time (min)**	**Velocity (mL/min)**	**A(%)**	**B(%)**
0	0.40	95	5
11.0	0.40	10	90
12.0	0.40	10	90
12.1	0.40	95	5
14.0	0.40	95	5

Supplement table-2. Conditions of positive and negative ion mode mass spectrometry agilent.

Parameter	ESI^+	ESI^-
Voltage	250	1500
Gas Flow	8	8
Fragmetor	135	135
Gas Temperature	325	325
Sheath Temperature	325	325
Sheath Flow	11	11
Nebulizer	40	40

Quality control samples (QC) were prepared by mixing sample extracts to analyze the repeatability of samples under the same processing method. In the process of instrumental analysis, a quality control sample was inserted into every 15 testing and analysis samples to monitor the repeatability of the analysis process.
